# Sargachromenol Isolated from *Sargassum horneri* Inhibits Particulate Matter-Induced Inflammation in Macrophages through Toll-like Receptor-Mediated Cell Signaling Pathways

**DOI:** 10.3390/md20010028

**Published:** 2021-12-24

**Authors:** D. P. Nagahawatta, Hyun-Soo Kim, Young-Heun Jee, Thilina U. Jayawardena, Ginnae Ahn, Jin Namgung, In-Kyu Yeo, K. K. Asanka Sanjeewa, You-Jin Jeon

**Affiliations:** 1 Department of Marine Life Sciences, Jeju National University, Jeju 690-756, Korea; dineth1673@gmail.com (D.P.N.); tuduwaka@gmail.com (T.U.J.); ikyeo99@jejunu.ac.kr (I.-K.Y.); 2 Department of Applied Research, National Marine Biodiversity Institute of Korea, 75, Jangsan-ro 101-gil, Janghang-eup, Seocheon 33662, Korea; gustn783@mabik.re.kr; 3 Department of Veterinary Medicine and Veterinary Medical Research Institute, Jeju National University, Jeju 690-756, Korea; yhjee@jejunu.ac.kr; 4 Department of Food Technology and Nutrition, Chonnam National University, Yeosu 59626, Korea; gnahn@chonnam.ac.kr; 5 Graduate School of Fisheries Sciences, Hokkaido University, 3-1-1 Minato, Hakodate, Hokkaido 041-8611, Japan; xmxmj@naver.com; 6 Department of Biosystems Technology, Faculty of Technology, University of Sri Jayewardenepura, Pitipana, Homagama 10206, Sri Lanka; 7 Marine Science Institute, Jeju National University, Jeju 63333, Korea

**Keywords:** Sargachromenol, *Sargassum horneri*, particulate matter, anti-inflammation, TLR

## Abstract

*Sargassum horneri* is an invasive brown seaweed that grows along the shallow coastal areas of the Korean peninsula, which are potentially harmful to fisheries and natural habitats in the areas where it is accumulated. Therefore, the author attempted to evaluate the anti-inflammatory mechanism of Sargachromenol isolated from *S. horneri* against particulate matter (PM)-stimulated RAW 264.7 macrophages. PM is a potent inducer of respiratory diseases such as lung dysfunctions and cancers. In the present study, the anti-inflammatory properties of Sargachromenol were validated using enzyme-linked immunosorbent assay (ELISA), Western blots, and RT-qPCR experiments. According to the results, Sargachromenol significantly downregulated the PM-induced proinflammatory cytokines, Prostaglandin E2 (PGE2), and Nitric Oxide (NO) secretion via blocking downstream activation of Toll-like receptor (TLR)-mediated nuclear factor kappa B (NF-κB) and MAPKs phosphorylation. Thus, Sargachromenol is a potential candidate for innovation in various fields including pharmaceuticals, cosmeceuticals, and functional food.

## 1. Introduction

Particulate matter (PM) causes various adverse respiratory problems such as decrease in lung functions and results in emergency hospital admissions or deaths [1]. As dust particles having a diameter less than 10 µm, they can penetrate the lungs and cause health issues such as lung cancers. Thus, PM is considered as one of the major air pollutants [2]. 

In Asia, extensive arid or semiarid highlands in northern China and Mongolia are the most important origins of dust [3]. Cyclone activities, their movements, and strong cold waves in these dust-generating areas play a major role in the formation of dust storms [4]. Microbiological materials and nitrates or sulfates derived from alkaline soil are the constituents of dust particles in these areas and have the potential to cause respiratory diseases [5]. Mortality in Central and East Asian countries is increased due to respiratory cardiovascular diseases through such dust storms [6]. 

Previous studies have reported that PM has a potential to induce inflammation in cells [7]. The cytokine production and elevated expression of inducible nitric oxide synthase (iNOS), cyclooxygenase-2 (COX-2), and other inflammatory genes indicate the inflammatory responses of cells. PM upregulates these inflammatory responses via the nuclear factor kappa B (NF-κB) and mitogen-activated protein kinase (MAPK) pathways. The MAPK pathway consists of extracellular signal-related kinases (ERK), c-jun N-terminal kinases (JNKs), and p38, which produce inflammatory cytokines and respond to external stimuli [8]. The activation of MAPK by PM plays a key role in tumor necrosis factor (TNF-α) production. Further, it activates NF-κB and regulates the expression of genes that are responsible for the production of cytokines, such as interleukin (IL)-1β and TNF-α [9]. Moreover, after exposing to PM, the p50 and p65 subunits of the NF-κB pathway are translocated from the cytoplasm to the nucleus, which causes the upregulation of NF-κB associated genes, such as IL-6 and TNF-α [7]. Toll-like receptors (TLRs) play a crucial role in immunity system by identifying pathogen associated molecular patterns from various outer stimulants. TLRs are categorized into two classes according to their localization, intracellular TLRs, and cell surface TLRs. The components of microbial membranes such as proteins, lipoproteins, and lipids can be recognized by the cell surface TLRs and intracellular TLRs mainly identified the nucleic acid derived from virus and bacteria. TLR mediated signaling consist of various adaptor proteins, directing to phosphorylation and activation in MAPK and NF-κB that results in generating immunity responses [10]. Therefore, identification of this signaling cascade and their regulation provide an insight into the manipulation of inflammatory responses.

Seaweeds are rich in polyphenols, polysaccharides, proteins, and vitamins; thus, numerous seaweed species are used in pharmaceutical, cosmeceutical, and food industries. Seaweeds have multiple bioactivities such as anti-inflammatory, anticancer, antioxidant, antibacterial, and anticoagulant activities [11,12]. Sargachromenol is a highly abundant sesquiterpene in *Sargassum horneri*. Terpenoids or its derivatives express anti-inflammation activities [13] and as a dietary bioactive compound, this reflects the beneficial consequences against chronic inflammation. In the present study, RAW 264.7 macrophages were used as an in vitro experimental model to determine the effect of Sargachromenol from *S. horneri* against PM-induced inflammation and the pathways to inhibit PM-stimulated inflammation including Toll-like receptors (TLRs), NF-κB, and MAPK pathways.

## 2. Results

### 2.1. Cell Viability 

**Figure 1:**
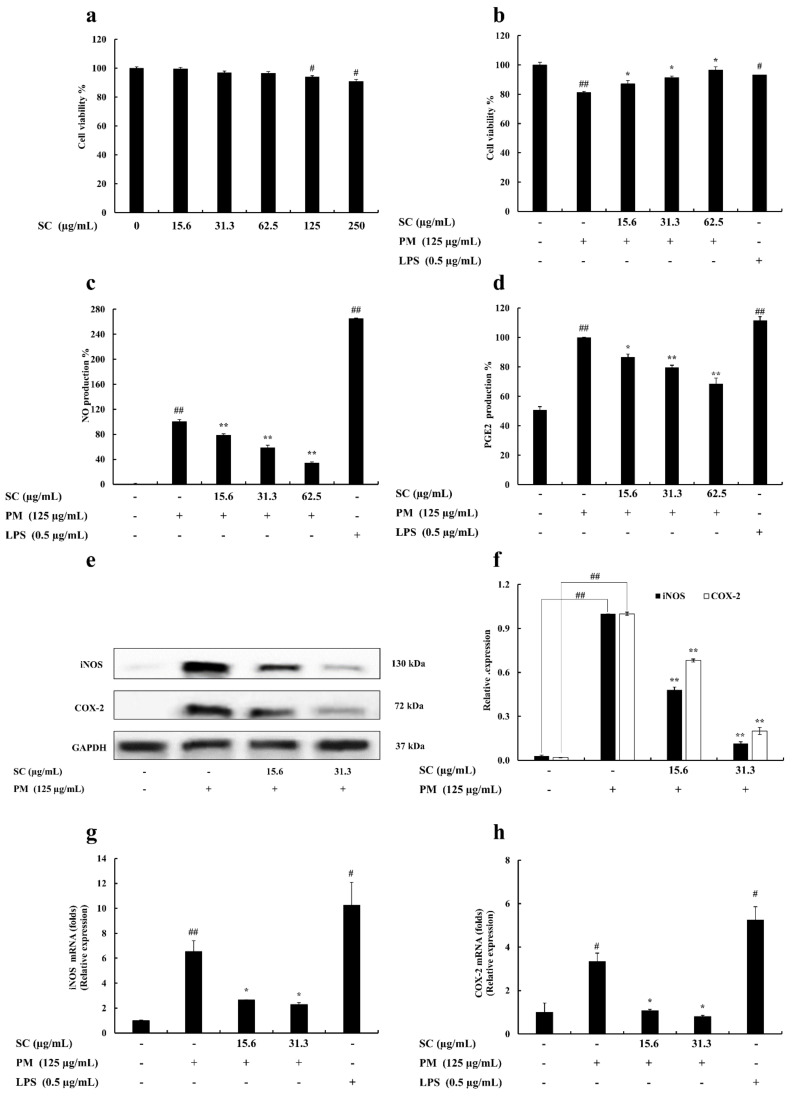
(**a**) Cytotoxicity, (**b**) cytoprotective, (**c**) nitric oxide (NO) inhibitory effect, and (**d**) prostaglandin E2 (PGE2) inhibitory effect of Sargachromenol (SC) in particulate matter (PM)-induced RAW 264.7 macrophages. Inhibitory effects of Sargachromenol (SC) on PM-induced inflammation associated proteins (**e**) inducible nitric oxide synthase (iNOS), Cyclooxygenase-2 (COX-2) protein expression, and (**f**) quantitative data. Glyceraldehyde 3-phosphate dehydrogenase (GAPDH) were used as internal controls. Western blot results were quantified using ImageJ software. Inflammation-associated gene expressions levels of (**g**) iNOS and (**h**) COX-2. Delta-Ct method was used to analyze the results of relative mRNA levels. The housekeeping GAPDH gene was used as an internal reference. Triplicated experiments were used to evaluate the data and the mean value is expressed with ± SD. * *p* < 0.05, ** *p* < 0.01, against PM treated group or # *p* < 0.05, ## *p* < 0.01, against control (ANOVA, Duncan’s multiple range test).

The effective concentration range (15.6, 31.3, 62.5, 125, and 250 μg/mL) of Sargachromenol was analyzed to discover the non-toxic concentrations on RAW 264.7 cells. The 15.6, 31.3, and 62.5 μg/mL concentrations did not exhibit any cytotoxic effect on RAW 264.7 macrophages (Figure 1a).

The selected concentrations of Sargachromenol were treated on PM-activated RAW 264.7 macrophages to evaluate the protective effect of Sargachromenol. According to the result, PM-stimulated RAW 264.7 cell viability was significantly decreased, but treatments of Sargachromenol significantly and dose-dependently reduced the PM-induced cytotoxicity and increased the cell viability (Figure 1b).

### 2.2. Determination of Nitric Oxide (NO) and Prostaglandin E2 (PGE2) Production

Griess assay was conducted to determine the NO prevention effect of Sargachromenol on PM-exposed RAW 264.7 macrophages. According to the results, PM upregulated the NO generation in RAW 264.7 macrophages. Selected Sargachromenol concentrations significantly declined the elevated NO generation in PM-stimulated cells in a dose-proportional manner (Figure 1c). The level of PGE2 in culture supernatant was determined using ELISA kits. Sargachromenol significantly prevented the secretion of PGE2 in PM-stimulated RAW 264.7 macrophages (Figure 1d).

### 2.3. Evaluation of Inducible Nitric Oxide Synthase (iNOS) and Cyclooxygenase-2 (COX-2) Levels in RAW 264.7 Macrophages

iNOS and COX-2 levels were evaluated to identify the mechanism underlying NO and PGE2 reduction following Sargachromenol treatment (15.6 and 31.3 μg/mL) in PM-stimulated (125 μg/mL) RAW 264.7 macrophages. The protein expression of COX-2 and iNOS was examined by Western blot and gene expression was examined by PCR. PM significantly increased the protein level and mRNA expression level of COX-2 and iNOS. Moreover, COX-2 and iNOS protein levels and gene expression levels in Sargachromenol-treated samples were significantly and dose-dependently downregulated (Figure 1e–h).

### 2.4. Evaluation of Cytokine Production

**Figure 2:**
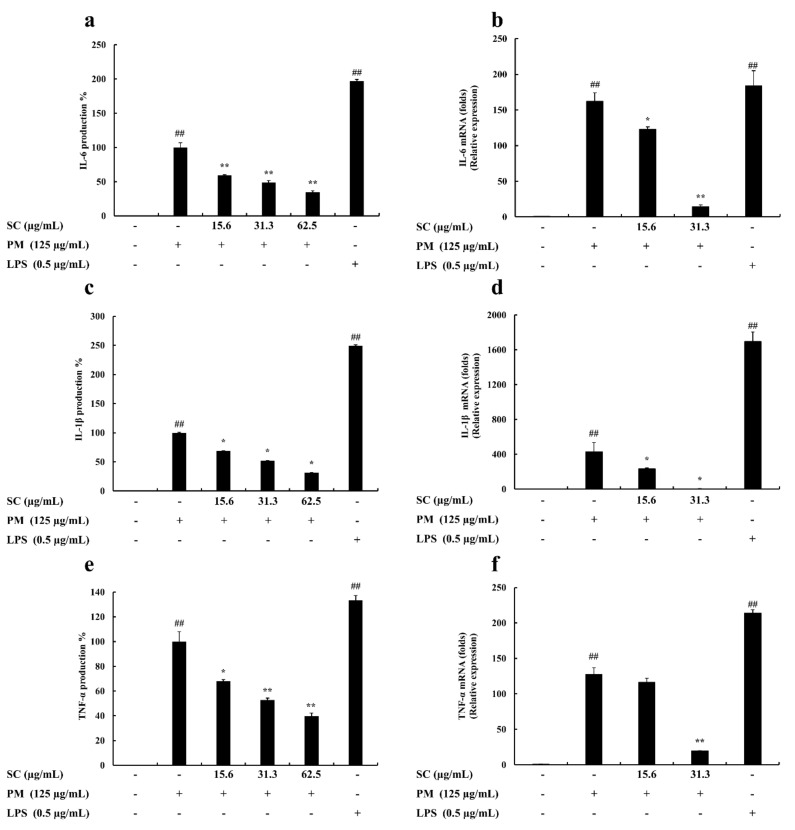
Inhibitory effects of Sargachromenol (SC) on particulate matter (PM)-induced inflammation associated proinflammatory cytokines production and gene expression in PM-induced RAW 264.7 macrophages. (**a**) Interleukin-6 (IL-6) cytokine, (**b**) IL-6 gene expression, (**c**) Interleukin-1β (IL-1β) cytokine, (**d**) IL-1β gene expression, (**e**) Tumor necrosis factor-α (TNF-α) cytokine, and (**f**) TNF-α gene expression. Proinflammatory cytokine production levels were evaluated using enzyme-linked immunosorbent assay (ELISA). Delta-Ct method was used to analyze the results of relative mRNA levels. The housekeeping glyceraldehyde 3-phosphate dehydrogenase (GAPDH) was used as an internal reference. Triplicated experiments were used to evaluate the data and the mean value is expressed with ± SD. * *p* < 0.05, ** *p* < 0.01, against PM treated group or ## *p* < 0.01, against control (ANOVA, Duncan’s multiple range test).

Proinflammatory cytokine (IL-1β, IL-6, and TNF-α) production in RAW 264.7 macrophages was evaluated using ELISA kits. The level of these cytokines decreased significantly and in a dose-proportional manner following Sargachromenol treatment (Figure 2). 

### 2.5. Gene Expression Levels Evaluation

**Figure 3:**
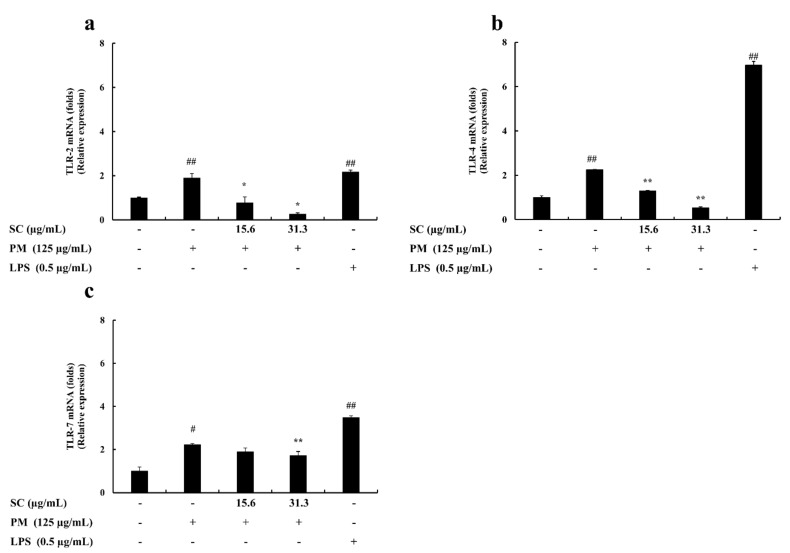
Protective effect of Sargachromenol (SC) against particulate matter (PM)-induced Toll-like receptor (TLRs) mRNA expressions. (**a**) TLR-2, (**b**) TLR-4, and (**c**) TLR-7. Delta-Ct method was used to analyze the results of relative mRNA levels. The housekeeping glyceraldehyde 3-phosphate dehydrogenase (GAPDH) was used as an internal reference. Triplicated experiments and trials. Results are expressed as the mean ± SD of three separate experiments. * *p* < 0.05, ** *p* < 0.01, against PM treated group or # *p* < 0.05, ## *p* < 0.01, against control (ANOVA, Duncan’s multiple range test).

qPCR was used to study the inhibitory effect of Sargachromenol on PM-stimulated RAW 264.7 macrophages. The mRNA expression of IL-6, IL-1β, TNF-α, TLR1~TLR9, iNOS, and COX-2 were measured to determine the level of inhibition. PM significantly induced the mRNA expression of IL-6, IL-1β, and TNF-α in PM-induced RAW macrophages. These results solidified the outcomes from ELISA evaluations. The results from the cytokine production analysis and mRNA expression analysis in PM-induced macrophages exhibited the same trend in the downregulation of PM-induced proinflammatory cytokine production. Among the mRNA expressions of TLRs in PM-induced RAW macrophages, TLR-2, TLR-4, and TLR-7 expressed a significant upregulation of their mRNA levels. However, Sargachromenol successfully downregulated these inclined expressions of TLRs in a significant and dose-dependent manner (Figure 2 and Figure 3). 

### 2.6. Western Blot Analysis

**Figure 4:**
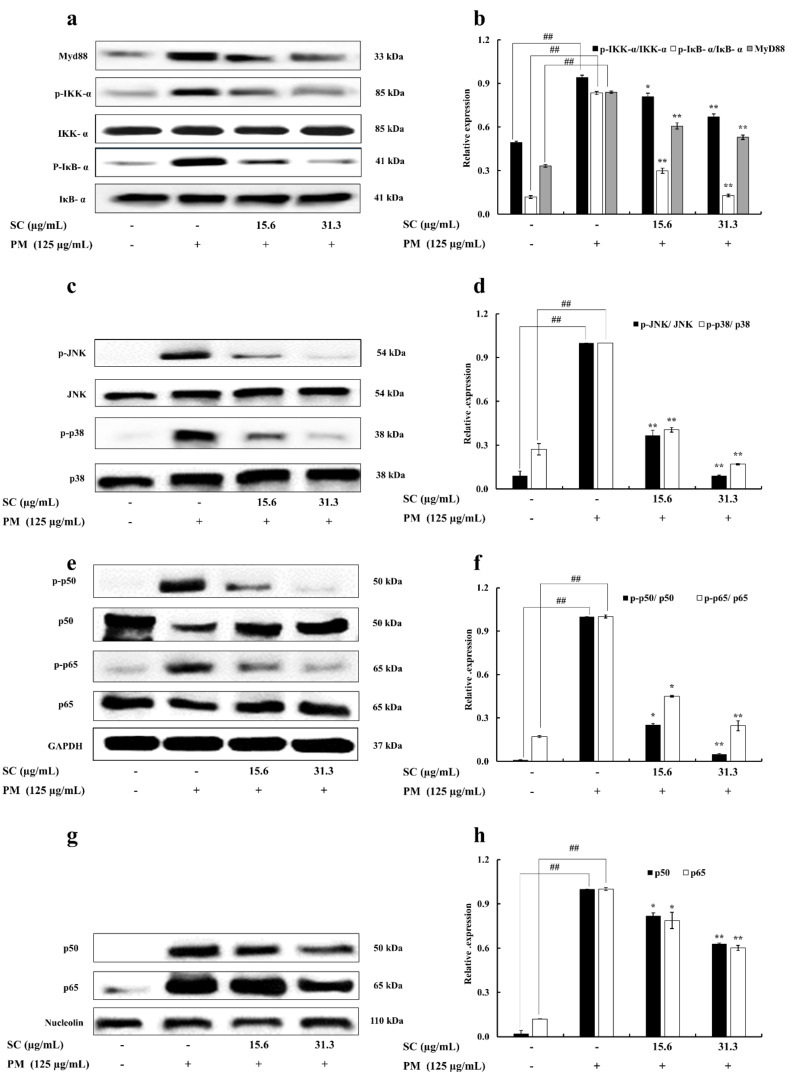
Inhibitory effects of Sargachromenol (SC) on particulate matter (PM)-induced Toll-like receptor (TLR)-mediated Nuclear factor κB (NF-κB) and Mitogen-activated protein kinase (MAPK) pathway associated protein expression in RAW 264.7 macrophage cells. (**a**) Myeloid differentiation primary response 88 (Myd88), inhibitor of NF-κB kinase α (IKK-α), and NF-κB inhibitory protein α (IκB-α), (**b**) quantitative data of Myd88, IKK-α, and IκB-α, (**c**) c-jun N-terminal kinase (JNK), p38, (**d**) quantitative data of JNK, p38, (**e**) p50 and p65 in cytoplasm, (**f**) quantitative data of p50 and p65 in cytoplasm, (**g**) p50 and p65 in nucleus, and (**h**) quantitative data of p50 and p65 in nucleus were determined using Western blotting. Glyceraldehyde 3-phosphate dehydrogenase (GAPDH) (for cytoplasm) and nucleolin (for nucleus) were used as internal controls. Western blot results were quantified using ImageJ software. Results are expressed as the mean ± SD of three separate experiments. * *p* < 0.05, ** *p* < 0.01, against PM treated group or ## *p* < 0.01, against control (ANOVA, Duncan’s multiple range test).

The effect of Sargachromenol on PM-stimulated RAW 264.7 macrophages was indicated by analyzing the activation of TLRs mediated NF-κB and MAPK signaling pathways. The protein expression of Myd88, an inhibitor of NF-κB kinase (IKK)-α, NF-κB inhibitory protein (IκB)-α and their phosphorylated forms were validated to determine the downstream activation process of TLRs. PM-significantly inclined the expression of Myd88 phosphorylation of IKK-α and IκB-α. This affects the downstream activation of NF-κB and MAPK signaling pathways. However, these protein expression results confirmed that treatments of Sargachromenol significantly downregulated the expression of Myd88 and phosphorylation of IKK-α and IκB-α. The phosphorylation of growth factor-regulated JNK and p38 proteins in PM-stimulated RAW 264.7 macrophages were measured to determine the effect of inhibition of Sargachromenol on the MAPK pathway. PM significantly increased the phosphorylation of JNK and p38 in PM-stimulated RAW macrophages. Further, treatments of Sargachromenol interestingly downregulated this elevated expression of the phosphorylation forms of JNK and p38. The protein expression of p65, p50, and their phosphorylated forms in the cytosolic and nuclear extract were evaluated to analyze the inhibitory effect of Sargachromenol on the NF-κB pathway. Here, the Western blot results of the NF-κB pathway revealed that PM significantly upregulated the phosphorylation and expression of p50 and p65 in the cytosol and nucleus, respectively. Sargachromenol significantly downregulated this upregulation and this solidifies the ability of Sargachromenol to downregulate the nuclear translocation of p50 and p65 in the NF-κB signaling pathway (Figure 4).

## 3. Discussion

Seaweeds are rich sources of nutrients as it possesses several useful bioactive compounds with anti-inflammation, antioxidant, anticoagulant, and antiviral properties [11,12]. Globally, there are approximately 150 species of Sargassum that have been identified in tropical, subtropical, and temperate areas, and it is a common macroalgae found along the coast of Japan and Korea [14]. In this study, we mainly targeted the anti-inflammatory activity of Sargachromenol, which is isolated from *S. horneri*, an edible brown seaweed in the Asia-Pacific region [15]. Our results indicated that Sargachromenol (15.6–62.5 μg/mL) showed low toxicity in RAW 264.7 macrophages and significantly increased the viability of PM-stimulated cells. This reflects the cytoprotective effect of Sargachromenol. Further, LPS was used as a reference in the present study to determine the activation of RAW 264.7 macrophages and produce inflammatory responses. 

Inflammation is a defensive mechanism against foreign biotic and abiotic factors such as bacteria and PM. It reduces the effects that exogenous factors induce in the body. Macrophages release defensive molecules to control or eliminate immune stimulants and increase the generation of proinflammatory mediators such as TNF-α, NO, IL-1β, IL-6, and PGE2 [16]. Thus, inflammation is a beneficial phenomenon in the body. However, excessive inflammatory responses can result in diseases such as septicemia, rheumatoid arthritis, hyperpyrexia, and cancer through acute and chronic inflammation [17].

Proinflammatory cytokines are produced in large amounts by activated macrophages. NO and PGE2 are proinflammatory mediators produced from iNOS and COX-2 proteins, respectively. The expression of iNOS and COX-2 results in the production of these proteins as an inflammatory response [18]. PGE2 is a dominant inflammation mediator in some conditions such as rheumatic arthritis and osteoarthritis. COX enzyme converts arachidonic acid into PGE2 in cells. Selective COX-2 inhibitors and nonsteroidal anti-inflammatory medications (NSAIDs) can be used to reduce inflammation in such conditions by modulating PGE2 production [19]. Excessive production of these mediators can cause proinflammatory diseases [20]. TNF-α can be considered as an ideal example of this. Local and systemic inflammation in patients with rheumatoid arthritis was effectively reduced by blocking TNF-α [21]. These inflammatory reactions and diseases can be prevented via the suppression of the aforementioned mediators [22]. 

According to the initial NO production analysis and ELISA results, Sargachromenol significantly downregulated the NO and PGE-2 production in PM-induced RAW macrophages. This revealed the potential of Sargachromenol for the regulation of proinflammatory mediators (NO and PGE-2). These outcomes were further confirmed by the results of Western blot and qPCR. According to them, Sargachromenol significantly declined the PM-induced gene and protein expression of iNOS and COX-2 that are responsible for producing NO and PGE-2, respectively. Moreover, the similar trend of PCR and Western blot results in significant downregulation of PM-induced iNOS and COX-2 expressions solidified the initial results. 

TLRs play an essential role in innate immunity and subsequent commencement of the adaptive immune response. Moreover, it stimulates inflammatory and antiviral responses. The cytoplasmic portion of the TLRs recruit various adaptor proteins and activate different transcription factors, such as NF-κB and MAPKs [23]. Signal transduction pathways, which are activated and initiated by TLRs, cause activation of various genes employed in host defense systems (chemokines, cytokines, and major histocompatibility complex) [24].

The stimulated TLRs activate the Ubc12/TAK1 pathway through myD88, IL-1 receptor-associated kinase (IRAks), and tumor necrosis factor receptor-associated factor 6 (TRAF6) [25]. When the receptor is induced, it recruits IRAK1, IRAK4, and TRAF6. IRAK1 is phosphorylated by IRAK4, is dissociated from the receptor with TRAF6, and is then interconnected with TGF-β-activated kinase 1 (TAK1) and TAK1-binding protein (TAB) 1, and TAB2. This complex forms a greater complex with Ubc13 and Uev1A and activates TAK1. The IKK complex (IKKα, IKKβ, IKKγ, and MAPK) is phosphorylated by activated TAK1 and causes the activation of transcription factor NF-κB and MAPK family members such as p38 and JNK that directly interfere with the regulation of inflammatory gene expression [26]. The activated NF-κB pathway translocates p65 and p50 from the cytoplasm to the nucleus via the phosphorylation of the IκB. NF-κB directs the expression of gene involved in cytokine production [23]. The present study showed, PM significantly stimulated the gene expression of TLR-2, TLR-4, and TLR-7 in RAW 264.7 macrophages. Meanwhile, Western blot results revealed that expression of Myd88, IKK-α, and IκB-α was also significantly upregulated with the PM treatment. This provides insight into the potential of PM for stimulating inflammatory signaling pathways. Nonetheless, Sargachromenol treatments in PM-induced macrophages significantly downregulated this protein expression in the cells. This leads to the regulation of protein expression in NF-κB and MAPK signaling pathways.

The dimeric transcription factor NF-κB belongs to the Rel-homology domain-containing protein family, whose members include p65, p50, p52, Rel B, and c-Rel. However, NF-κB is considered as a heterodimer comprising p65 and p50. This heterodimer has two forms as activated and inactivated. Usually, it is inactivated in non-stimulated cells by interacting with an inhibitor of NF-κB proteins [23]. The NF-κB pathway can be activated by excessive generation of proinflammatory cytokines (IL-6, IL-1β, and TNF-α) and the expression of inflammation-related genes (iNOS and COX-2). The NF-κB transcription factor has a major role in inflammation and innate immunity responses [27]. Moreover, NF-κB transcription factor not only acts as a regulator of inflammation but also acts as a regulator of apoptosis. Because of this, the inactivation of NF-κB transcription factor strongly affects inflammatory responses in macrophages [28]. This indicates a possible way to control abnormal inflammatory responses. The results from the Western blot in the present study revealed that Sargachromenol significantly and dose-dependently downregulated the PM-induced phosphorylation of p50 and p65 NF-κB subunits in the cytosol of the macrophage. Further, expression of p50 and p65 in the nucleus was also significantly downregulated with the Sargachromenol treatments followed by a similar trend. Altogether, results of TLRs, Myd88, IKK-α, IκB-α, p50, and p65 revealed the potential of Sargachromenol in the PM-induced inflammatory response generation.

Considering the previous studies, MAPK pathway plays a significant role in inflammation. MAPK turns extracellular signals and causes the activation of intracellular signaling pathways through TLR-mediated Myd88-dependent signaling pathway. This was confirmed by the results that Tab protein-deficient mice did not exhibit any abnormalities in TLR signaling pathway. [29]. MAPK belongs to a signal transduction protein family and is conserved not only in multicellular organisms but also in yeast. There are several subfamilies in MAPKs; among these, JNKs and p38 isoforms (p38s) were evaluated in this study [15,30,31]. JNK has more than 10 isoforms, which are transcribed from three genes: JNK1, JNK2, and JNK3 [31]. JNK1 and JNK2 are expressed in all cell types in the body, and JNK3 is mainly found in the brain [32]. In mammals, p38 has four isoforms: p38α, p38β, p38γ, and p38δ. Further, p38α is a serine/threonine kinase and has the ability to respond expeditiously to different kinds of stresses [33]. Moreover, p38 plays a pivotal role in inflammation and not only regulates the production of leading inflammatory mediators but also can reduce the level of cytokines (TNF-α) [34]. The Western blot results in the current study revealed that Sargachromenol significantly downregulated the PM-induced phosphorylation of p38 and JNK in RAW macrophages in a similar trend with TLRs Myd88. This provides an insight into the anti-inflammatory activity of Sargachromenol through TLR-mediated MAPK signaling. 

The results from the ELISA assay revealed that Sargachromenol significantly and dose-dependently downregulated the PM-induced proinflammatory cytokine production (TNF-α, IL-1β, and IL-6). This further solidified all the mRNA expression and Western blot results related to the mentioned proinflammatory cytokines, NF-κB, and MAPK signaling pathways. Overall, these observations verify the potential of Sargachromenol against chronic inflammatory conditions and this study encourages further studies to confirm the relationship of anti-inflammatory activity of Sargachromenol through TLR-mediated NF-κB and MAPK signaling pathways using pharmacological inhibitors.

## 4. Materials and Methods

### 4.1. Materials

*S. horneri* was collected from January 2017 to May 2017 along the shores of Jeju Island, Korea. The collected samples were cleaned under running water to detach the sand, salt, and, attached epiphytes. Washed seaweeds were freeze dried and ground to make a powder. It was filtered through 10–80 mesh size sieve. The prepared *S. horneri* was stored in a 20 °C refrigerator.

### 4.2. Chemicals and Reagents

RAW 264.7 macrophages were obtained from the Korean Cell Line Bank (KCLB), Seoul, Korea. The growth medium Dulbecco’s modified Eagle’s medium (DMEM), fetal bovine serum (FBS), and penicillin-streptomycin were obtained from Gibco BRL (Burlington, Canada). Enzyme-linked immunosorbent assay (ELISA) kits for mouse TNF-α, IL-1β, IL-6, and prostaglandin E2 (PGE2) were obtained from R & D system (Minneapolis, MN, USA). TRI Reagent, 3-(4,5-dimethylthiazol-2-yl)-2,5-diphenyltetrazolium bromide (MTT), and dimethyl sulfoxide (DMSO) were obtained from Sigma-Aldrich (St. Louis, MO, USA). Antibodies used in Western blot (iNOS, COX-2, MyD88, IKK-α, p-IKK-α, IκB-α, p-IκB-α, p65, p-p65, p50, p-p50, p38, p-p38, JNK, and p-JNK) were obtained from Santa Cruz Biotechnology (Santa Cruz, CA, USA).

### 4.3. Preparation of 70% Ethanol Extract and Compound Isolation

**Figure 5:**
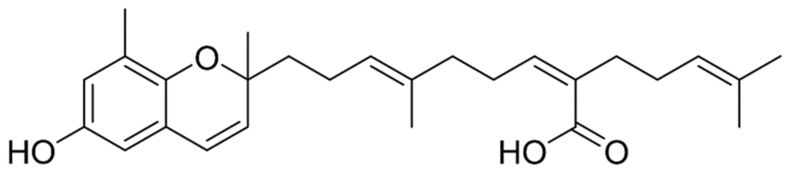
Molecular structure of Sargachromenol (SC).

*S. horneri* was air dried at 45–55 °C and ground into fine particles. The ground *S. horneri* was filtered using pin-mill mesh (40–50 μm). The *S. horneri* powder was extracted using 70% ethanol for 12 h at 65–80 °C. The extract was concentrated and freeze dried. Then, the 70% ethanol extract powder was dissolved in pure ethanol solution, and the resulting solution was mixed with white clay for 2 h to eliminate residues. Then, the mixture was centrifuged at 22,337× *g* at room temperature. Pellet was discarded, the supernatant was concentrated and mixed with pure ethanol. This solution was mixed with cellulose to remove heavy metals followed by centrifugation under the aforementioned conditions. Then, the concentrated supernatant was mixed with 95% ethanol and evaporated to increase its purity, and this resulting powder was considered as *S. horneri* ethanol extract (SHE) [35]. The crude SHE was prepared and provided by Seojin Biotech Co. Ltd., Korea (lot number SJFC70180625). The consequent powder of SHE was used for further purifications to isolate Sargachromenol with 90% purity (C27H36O4). Briefly, n-hexane and ethyl acetate were utilized to partition SHE according to its polarity. The n-hexane fraction of SHE (SHEH) was separated further using ODS open column chromatography following n-hexane: ethyl acetate ratio (F1) 90:10, (F2) 80:20, (F3) 70:30, (F4), 64:40, and (F5) 50:50. Preparative HPLC was utilized to further purification of F4. The reverse-phase HPLC, YL900 HPLC system (YL9120 UV/Vis detector) with Cosmosil, 10 µm, 10 × 250 mm semi-preparative C18 column was used for further purification. Distilled water with 0.01% trifluoroacetic acid (A) and acetonitrile with 0.01% trifluoroacetic acid (B) were the mobile phase. The high performance liquid chromatography (HPLC) system equipped with a PDA detector (Waters Corporation, Milford, MA, USA) coupled with electro spray ionizing mass spectrometer (HPLC-DAD-ESI/MS, Hewlett-Packard, Waldbronn, Germany) was utilized to determine the mass of isolated compound. The molecular ion peak was observed at 424.23 g/mol and this agrees with the theoretical molecular weight 424.6 g/mol of Sargachromenol. Further, the fragment pattern and relevant fragments were matched well with sample library of the Natural Medicine Research Center, Korea Research Institute of Bioscience and Biotechnology (KRIBB, Chungcheongbuk-do, Korea). Nuclear magnetic resonance (NMR) spectroscopy was conducted to elucidation of Sargachromenol structure (400 MHz, JNM-ECX400, JEOL, Japan). 1H (CD3OD, 400 MHz) and 13C (CD3OD, 100 MHz) spectra were obtained [36] (Figure 5).

### 4.4. Cell Culture and Sample Preparation

DMEM (high glucose) with 10% FBS (heat inactivated) and 1% penicillin/streptomycin antibiotics was used as a growth media for RAW 264.7 macrophages. Cultured cells were kept in a controlled environment at 37 °C temperature with 5% CO_2_. Isolated Sargachromenol was dissolved in DMSO and diluted in DMEM for treating cells. Final DMSO concentration of each treatment was <1%. 

### 4.5. Dose Range Determination

The cytotoxicity of Sargachromenol in RAW 264.7 macrophages was evaluated using previously described colorimetric MTT assay with slight modifications [37]. Briefly, 96-well plates were used to seed cells (1 × 10^5^ cells/mL) followed by incubation for 24 h in dark condition under 37 °C temperature with 5% CO_2_. Subsequently, cells were treated with different concentrations of Sargachromenol (15.6, 31.3, 62.5, 125, and 250 μg/mL) for 24 h. Each well was treated with MTT solution prepared in phosphate-buffered saline (200 μL per well). After 2 h of incubation, formazan crystals were formed and dissolved in DMSO. Absorbance was assessed at 540 nm using a micro-plate reader (Synergy^TM^ HT, Winooski, VT, USA). For calculation, PM-untreated cells were considered to have 100% cell viability. Absorbance values corresponding to MTT reduction in treated cells were expressed as mean percentage values relative to control cells.

### 4.6. Determination of Cell Viability

MTT assay was used to detect the viability of Sargachromenol-treated (15.6, 31.3, and 62.5 μg/mL) PM-stimulated RAW 264.7 macrophages. The cells concentration of each well was 1 × 10^5^ cell/mL and seeded cells were incubated (dark condition, 37 °C, 5% CO_2_) for 24 h, and were treated with Sargachromenol. After 1 h, cells were treated with PM (125 μg/mL) and incubated for 23 h. Then, each well was treated by MTT (200 μg). The formed formazan crystals were dissolved in DMSO after 1 h. The dissolved formazan was quantified using a spectrophotometer at 540 nm (BioTek Instruments, Winooski, VT, USA). The absorbance values were expressed as mean percentage values relative to control cells [37].

### 4.7. Determination of Nitric Oxide (NO) Production 

Griess assay was used to evaluate the NO inhibition ability of Sargachromenol in PM-stimulated RAW 264.7 macrophages. Briefly, 24-well cell culture plates were used to seed the cells (1 × 10^5^ cells/mL) and Sargachromenol was added after a 24 h period of incubation. Then, PM was added after a 1 h incubation period and incubation was continued for another 24 h. Then, 50 μL of Griess reagent and culture supernatant was mixed together and the absorbance values were measured at 540 nm after 10 min incubation period [38]. Absorbance values of the treated cells were expressed as mean percentage values relative to control cells.

### 4.8. Evaluation of the Production of Cytokines and PGE2

The ELISA kits were used with manufacturer’s instructions to evaluate the inhibitory activity of Sargachromenol on cytokines (IL-1β, IL-6, and TNF-α) and PGE2 production. RAW 264.7 macrophages were seeded and treated by selected Sargachromenol concentrations (15.6, 31.3, and 62.5 μg/mL) in the existence or absence of PM (125 μg/mL) for 24 h; then, cell culture supernatants were used to analyze the extent of proinflammatory cytokines levels.

### 4.9. Western Blot Analysis

Six-well plates were used to seed cells (1 × 10^5^ cells/mL) and incubated for 24 h. Then, Sargachromenol treated cells were stimulated by PM. Protein extraction from the cells was conducted using NE-PER^®^ Nuclear and Cytoplasmic Extraction Kit (Thermo Scientific, Rockford, IL, USA) based on a previously described method [39]. Cells were harvested based on the protein to be analyzed. The expression of proteins in the NF-κB and MAPK pathways was analyzed by harvesting cells within 20 min of PM stimulation and iNOS and COX-2 after 24 h of PM stimulation [40]. Moreover, nitrocellulose membrane and sodium dodecyl sulfate-polyacrylamide gel electrophoresis (12%) were used to immobilize the proteins by electrotransferring an equivalent amount of proteins onto the membrane. 

Non-fat milk (5%) was used as a blocking solution (60 min); nitrocellulose membrane was incubated overnight with selected primary antibodies (iNOS, COX-2, MyD88, IKK-α, p-IKK-α, IκB-α, p-IκB-α, p65, p-p65, p50, p-p50, p38, p-p38, JNK, and p-JNK) in 5% skimmed milk. Membrane was washed with Tween 20/Tris-buffer saline (3 times), and horseradish peroxidase-conjugated secondary antibody (anti-mouse or anti-rabbit) was loaded onto the membrane, followed by incubation for 2 h. The chemiluminescence substrate (Amersham, Arlington Heights, IL, USA) was used to improve signals, and ImageJ 1.49v (National Institutes of Health, Bethesda, MD, USA) was used to analyze the expressions.

### 4.10. Total RNA Extraction and cDNA Production

Total RNA extraction was performed using TRIzol reagent according to manufacturer’s instructions. A micro drop plate (Thermo Scientific, Waltham, MA, USA) was used to determine purity and concentration. Diluted RNA sample (1 µg/µL) was used to produce cDNA using a first-strand cDNA synthesis kit (TaKaRa, Tokyo, Japan).

### 4.11. Quantitative Real-Time Polymerase Chain Reaction (qPCR) Analysis

Proinflammatory cytokine gene expressions was analyzed using SYBR Green qPCR using a Thermal Cycler Dice-Real Time System (TaKaRa). GAPDH was used as the internal standard. The thermal profile was as below: 10 s at 95 °C, 40 cycles of 5 s at 95 °C; 10 s at 55 °C; 20 s at 72 °C; 15 s at 95 °C; 30 s at 55 °C; and: 15 s at 95 °C [41]. Table 1 summarizes the primers used for this study.

### 4.12. Statistical Analysis

All experiment results were expressed as the mean value with the standard deviation of three independent recurrences. The significant differences of all statistical data were analyzed via one-way analysis of variance according to Duncan’s multiple range test using the SPSS v. 20 (IBM Corp., Armonk, NY, USA).

## 5. Conclusions

In summary, the present study provides an insight into PM-induced inflammation and its reduction by Sargachromenol. The inflammatory end product levels were decreased by regulating the expression of iNOS, COX-2, TNF-α, IL-1β, and IL-6. Moreover, this was supported by controlling the signal transduction of TLR, NF-κB, and MAPK pathways by Sargachromenol. Thus, the results strongly suggest that Sargachromenol can abate PM-stimulated inflammation in RAW 264.7 macrophages and upregulate cellular resistance. Further, Sargachromenol is a potential candidate to be developed for application in food chemistry, medical and pharmaceutical sectors, and could be a potential marine natural product in the mentioned fields. 

## Figures and Tables

**Table 1:** Primers used in the present study.

Gene	Primer	Sequence
GAPDH	Sense	5′-AAGGGTCATCATCTCTGCCC-3′
	Antisense	5′-GTGATGGCATGGACTGTGGT-3′
iNOS	Sense	5′-ATGTCCGAAGCAAACATCAC-3′
	Antisense	5′-TAATGTCCAGGAAGTAGGTG-3′
COX-2	Sense	5′-CAGCAAATCCTTGCTGTTCC-3′
	Antisense	5′-TGGGCAAAGAATGCAAACATC-3′
IL-6	Sense	5′-GTACTCCAGAAGACCAGAGG-3′
	Antisense	5′-TGCTGGTGACAACCACGGCC-3′
IL-1β	Sense	5′-CAGGATGAGGACATGAGCACC-3′
	Antisense	5′-CTCTGCAGACTCAAACTCCAC-3′
TNF-α	Sense	5′-TTGACCTCAGCGCTGAGTTG-3′
	Antisense	5′-CCTGTAGCCCACGTCGTAGC-3′
TLR-2	Sense	5′-CAGCTGGAGAACTCTGACCC-3′
	Antisense	5′-CAAAGAGCCTGAAGTGGGAG-3′
TLR-4	Sense	5′-CAACATCATCCAGGAAGGC-3
	Antisense	5′-GAAGGCGATACAATTCCACC-3′
TLR-7	Sense	5′-TTCCTTCCGTAGGCTGAACC-3′
	Antisense	5′-GTAAGCTGGATGGCAGATCC-3′

Glyceraldehyde 3-phosphate dehydrogenase (GAPDH), inducible nitric oxide synthase (iNOS), cyclooxygenase-2 (COX-2), interleukin-6 (IL-6), interleukin-1β (IL-1β), tumor necrosis factor-α (TNF-α), TLR-2 (Toll-like receptor-2), TLR-4 (Toll-like receptor-4), TLR-7 (Toll-like receptor-7).
